# Paleoproterozoic Mississippi Valley-type mineralization at Black Angel, Greenland: evidence from sulfide δ^66^Zn and rhenium-osmium geochronology

**DOI:** 10.1007/s00126-024-01332-w

**Published:** 2024-12-09

**Authors:** Nicolas J. Saintilan, Corey Archer, Kristoffer Szilas, Kristina Krüger Geertsen, Diogo Rosa, Jorge E. Spangenberg

**Affiliations:** 1 Institute of Geochemistry and Petrology, Department of Earth and Planetary Sciences, ETH Zürich, Zürich, Switzerland; 2 Department of Geosciences and Natural Resource Management, University of Copenhagen, Copenhagen, Denmark; 3 Department of Mapping and Mineral Resources, Geological Survey of Denmark and Greenland (GEUS), Copenhagen, Denmark; 4 Institute of Earth Surface Dynamics, University of Lausanne, Lausanne, Switzerland; 5 Present Address: Department of Geological Sciences, University of Alabama, Box 870338, Tuscaloosa, AL 35487 USA

## Abstract

We provide timestamps for the major zinc-lead (Zn-Pb) Mississippi Valley-type Black Angel deposit (Greenland) based on new pyrite rhenium-osmium (Re-Os) isotope geochemistry data: (1) a Re-Os isochron age 1,884 ± 35 million years ago (Ma – 2σ, 1.8%) for subhedral pyrite cemented by sphalerite ± galena in dolomitized clean limestone, and, (2) a Re-Os model age 1,828 ± 16 Ma (2σ, 0.9%) for epigenetic massive pyrite in siltstone/mudstone cap rock. Zinc-lead mineralization in evaporite-bearing carbonates in the Karrat Basin took place ca. 1,884 Ma at the time of far-field fluid flow associated with back-arc spreading ca. 1,900–1,850 Ma. Mineralization predates the development of the Rinkian foreland basin (ca. 1,850 – < 1,800 Ma) and a collisional stage (ca. 1,830 – < 1,800 Ma) in the context of the telescoping Rinkian and the Nagssugtoqidian Orogens. Replacement of clean carbonate and sustained acid neutralization led to significant sphalerite precipitation ca. 1,884 Ma. Conversely, precipitation of epigenetic massive pyrite in the cap rock ca. 1,828 Ma may signal (1) the lack of chemical reactivity of the cap rock for the pH-buffered conditions needed for Zn-Pb mineralization, and (2) the unfavorable impact of incipient regional Rinkian metamorphism (ca. 1,830–1,800 Ma) and tectonic compression on aquifer permeability and continued brine migration. The initial ^187^Os/^188^Os ratio (Os_i-pyrite_ = 1.07 ± 0.32) from isochron regression identifies a crustal origin for Os and, by corollary, other metals in the ca. 1,884 Ma Zn-Pb mineralization. Although the Rae Craton basement rocks comprise the dominant source for metals (based on our Os_i-pyrite_ and δ^66^Zn_pyrite/sphalerite_ data), we identify a complementary contribution in Zn (maximum 12–24%) from Paleoproterozoic sedimentary carbonate. This source of Zn in sedimentary calcite is deemed possible in the context of Paleoproterozoic seawater at high Na/Cl ratio and in the absence of Zn-based eukaryotic metabolism in shallow marine environment.

**Supplementary Information:**

The online version contains supplementary material available at 10.1007/s00126-024-01332-w.

## Introduction

**Fig. 1:**
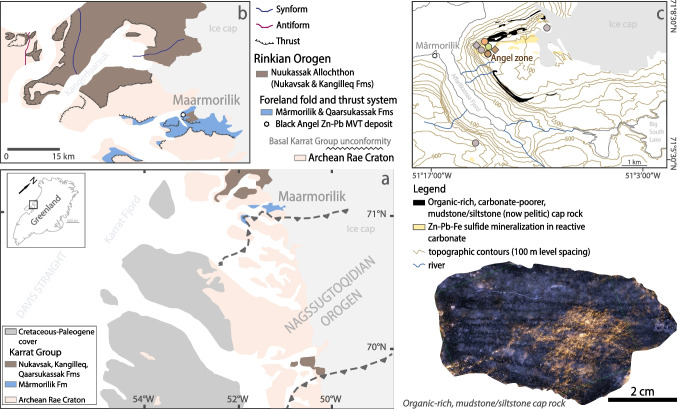
**a**. Regional geological map of the Karrat Group around the Maarmorilik area in West Greenland. **b**. Close-up on the Maarmorilik area and the discordant position of several thrust sheets of the Rinkian Orogen and its telescoping with the Nagssugtoqidian Orogen (after Rosa et al. 2023; Guarnieri et al. 2023). **c**. Topographic map with geological extent of Zn-Pb-Fe sulfide mineralization at Black Angel versus pelitic, possibly graphitic, carbonate-poor, mudstone cap rock. The location of the samples utilized in the present work is shown (diamonds: sphalerite samples; circle: pyrite samples. For color codes, see Fig. 5). Inset shows an example of the organic-rich, carbonate-poorer mudstone/siltstone (now metamorphosed to pelite) cap rock at Black Angel

According to the widely accepted model for Phanerozoic Mississippi Valley-type (MVT) deposits (Leach et al. 2010), the occurrence of MVT deposits in the geological record is intimately linked to the oxygenation of Earth’s hydrosphere, the development of platform carbonates in passive margin settings, and sufficient seawater sulfate (> 2.5 mM [millimolar] SO_4_^2−^; Algeo et al. 2015) to contribute hydrogen sulfide to fix metals in favourable trap sites that locate the reduction of seawater sulfate (Kesler and Reich 2006; Kesler et al. 2007; Leach et al. 2010). Carbonate-hosted MVT deposits remained scarce in the rock record until the Neoproterozoic Oxygenation Event (NOE), with the exception of: (i) the possibly oldest ca. 2,050–2,020 million-year-old (myr-old) Pering and Bushy Park deposits in South Africa (Gutzmer 2006; Huizenga et al. 2006), and (ii) the assumed ca. 1,860–1,830 myr-old Black Angel deposit in Central West Greenland (Fig. 1; Rosa et al. 2023). During the NOE, the oceans were further oxygenated (Fike et al. 2006; Sahoo et al. 2012; Williams et al. 2019). After the NOE, the seawater sulfate reservoir could be maintained at mM levels, similar to the Phanerozoic (Algeo et al. 2015), i.e., a paramount requirement for district-scale MVT mineralization (Kesler et al. 2007). In addition to the critical role of the redox state of the sulfur cycle in seawater prior to the NOE, Leach et al. (2010) suggested that the lithology and reactivity of the carbonate platforms also limited the formation of MVT deposits during the Proterozoic, i.e., low mineralizing potential for MVT deposits in Proterozoic, fine-grained, stromatolitic carbonate mudstones (Lucia 2007) versus higher mineralizing potential for MVT deposits in abundant permeable carbonates with coarse, skeletal carbonate grains and fragments from the late Neoproterozoic onwards (Hazen et al. 2008 and references therein). These lithological and geochemical parameters highlight the fact that a comprehensive temporal record of pre-Phanerozoic MVT deposits is critically needed (see Leach et al. 2010) to propose genetic models for pre-Phanerozoic MVT deposits. Yet, this framework is missing due to the absence of absolute radiometric ages of ore minerals despite primary features of epigenetic sediment-hosted deposits, which are needed for such investigations, surviving even at high metamorphic grades in the rock record (see Leach et al. 2010).

**Fig. 2:**
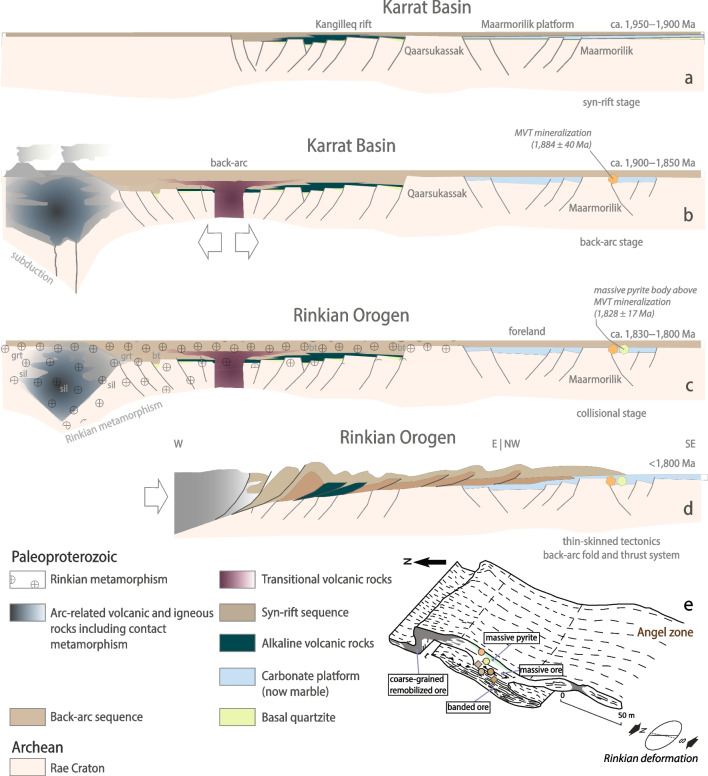
**a** to **d**. Paleoproterozoic evolution of the Karrat Basin and evolution of the Rinkian Orogen between ca. 1,950 to < 1,800 Ma (after Guarnieri et al. 2023). The location and timing of MVT mineralization at Black Angel according to our new pyrite Re-Os isochron age are shown in panels **b**, **c**, **d** & **e**. Three-dimensional representation of the Angel orebody (from Rosa et al. 2023; modified from Pedersen 1980) with location of the samples utilized in the present work (diamonds: sphalerite samples; circle: pyrite samples. For color codes, see Fig. 5). Abbreviations: grt: garnet, sil: sillimanite, bt: biotite

To address these questions, we use the case study of the Black Angel zinc-lead (Zn-Pb) deposit (pre-mining reserves: 13 million metric tons at 12 wt.% Zn, 4 wt.% Pb, and 29 parts per million – ppm Ag; Thomassen 1991) that is hosted in a carbonate-evaporite platform of Paleoproterozoic age and is interpreted as a MVT deposit (Rosa et al. 2023). In the context of the generally accepted low level of marine sulfate (2.5 mM ≥ SO_4_^2−^ > 200 µM) in the Paleoproterozoic (Farquhar et al. 2010) in the aftermath of the Great Oxidation Event (GOE – ca. 2,460 to 2,426 million years ago – Ma; Gumsley et al. 2017) and the Lomagundi Carbon Excursion (ca. 2,220 to 2,060 Ma; Karhu and Holland 1996; Martin et al. 2013), the ca. < 2,000–1,950 Ma Maarmorilik carbonate platform in the Karrat Group of West Greenland stands out as the last known sulfate-rich evaporite-carbonate sequence until the Mesoproterozoic (Figs. 1 and 2; Rosa et al. 2023). The building of the extensive Maarmorilik mixed-sulfate-evaporite-carbonate platform generated significant volumes of highly saline fluids in a “brine factory” (Leach et al. 2010) that triggered large-scale albitization of Archean Rae Craton basement rocks as well as Paleoproterozoic sedimentary rocks (Kalsbeek 1992; Ryan and Escher 1999; Rosa et al. 2023). This geological context provided the ground preparation and the ingredients for hydrothermal MVT mineralizing processes at Black Angel (Rosa et al. 2023). Here, we constrain a temporal and geodynamic context for mineralization mechanisms that are compatible with a MVT genetic model. To reach this conclusion, we document the absolute timing of Zn-Pb MVT mineralization at Black Angel using pyrite rhenium-osmium (Re-Os) isotope geochemistry. Then, by using sphalerite and pyrite Zn stable isotope geochemistry (δ^66^Zn_JMC-Lyon_), we explore the role and reactivity of Paleoproterozoic sedimentary carbonate to control (1) the modal proportions of sulfide species in the ore and (2) the extent of economic Zn mineralization in the assumed unfavourable geological context of carbonate platforms dominated by fine-grained, stromatolitic mudstone in the Paleoproterozoic. Our work highlights that, aside from the commonly accepted and here confirmed main metal source in basement rocks, a complementary metal source for MVT mineralization may exist in Paleoproterozoic carbonates. Our study also substantitiates the conclusions by Leach et al. (2005, 2010) that the economic potential for MVT deposits in Proterozoic carbonates depends on the extent of sphalerite precipitation that is controlled by the reactivity of carbonate lithologies for acid neutralization (see Liu et al. 2021).

## Geological setting

### Geodynamic evolution 2,000–1,800 Ma

The Paleoproterozoic Mârmorilik Formation in the Karrat Basin of West Greenland hosts the Black Angel Zn–Pb deposit (Fig. 1). We summarize the geodynamic evolution of the area of the Maarmorilik evaporite-carbonate platform during the interfering Paleoproterozoic Rinkian and Nagssugtoqidian Orogens (Fig. 1; after Guarnieri et al. 2023). The Mârmorilik Formation is part of the Paleoproterozoic (< 2,000–1,850 Ma) Karrat Group (Figs. 1 and 2a) that was deposited as a result of stepwise crustal extensional tectonics evolving from (1) a < 2,000 to 1,900 Ma synrift stage accompanied by alkaline volcanism (Fig. 2a), to (2) a post-1,900 Ma back-arc stage coeval with basin development in the upper plate above an eastward-subducting plate (Fig. 2b, c).

#### Rifting stage (< 2,000–1,900 Ma)

The Qaarsukassak Formation (basal Karrat Group), which comprises quartz-rich mature sediments and unconformably overlies the Qeqertarssuaq complex and Alfred Wegener Halvø Complex of the Archean Rae Craton, probably filled a sag basin (Guarnieri et al. 2023). Paleoproterozoic detrital zircon grains in the Qaarsukassak Formation signal a maximum depositional age ca. 2,000 Ma. The development of this sag basin was followed by intraplate basaltic volcanism (oceanic-island-basalt-type affinity) heralding the rifting stage of the Karrat Basin (Fig. 2a; Guarnieri et al. 2022a, 2023). This stage is coeval with deposition of synrift siliciclastic rocks of the lower Nûkavsak Formation that are, at their base, interbedded with mafic volcanic rocks. The central part of the basin was defined by the volcanic rift and separated from the platform by a structural high only covered by quartzites (Guarnieri and Baker 2022). In the south, around Maarmorilik, a quartzitic member was deposited on gneisses of the Archean Rae Craton, prior to the building of an evaporite-carbonate platform. Detrital zircons from siliciclastic layers intercalated with dolomitic carbonates (now present as marbles) indicate a minimum depositional age ca. 1,915 Ma (Guarnieri et al. 2022a). New tectono-stratigraphic evaluation constrains a possible depositional age of the Mârmorilik Formation between 1,950 and 1,900 Ma (Guarnieri et al. 2023, their Fig. 16). Permeability channels for hydrothermal fluid flow existed, in particular in the Maarmorilik area, at the interface between rift-wide listric normal faults and transfer faults, with NNE-SSW– and ESE-WNW–trends, respectively (Guarnieri and Baker 2022; Guarnieri et al. 2023).

#### Back-arc stage (1,900–1,850 Ma)

The back-arc stage (Fig. 2b) reflects the evolution of the Rae craton continental margin in the upper plate, beginning with subduction, which led to arc magmatism and eventually the intrusion of the Prøven igneous complex between ca. 1,900 Ma and 1,850 Ma. In the south at Maarmorilik, back-arc extensional tectonics led to the drowning of the evaporite-carbonate platform covered by siliciclastic sediments (Guarnieri et al. 2023).

#### Compressional tectonics of the Rinkian Orogen (1,830–1,800 Ma) and later Nagssugtoqidian deformation

The collisional phase of the Rinkian Orogeny (ca. 1,900 – 1,800 Ma; Fig. 2c, d) took place ca. 1,830–1,800 Ma and led to metamorphism of the Karrat Group and magmatic arc rocks (Kirkland et al. 2017; Guarnieri et al. 2022b). This geodynamic configuration explains why the metamorphic grade of the Karrat Group increases from greenschist facies in the south (Maarmorilik in foreland setting) to granulite facies in the north (back-arc setting), including migmatitization and emplacement of S-type leucogranites. After 1,800 Ma, the Rinkian Orogeny culminated in thin-skinned tectonics in a back-arc fold and thrust system. Late collisional events resulted from the interference between the Rinkian and Nagssugtoqidian (ca. 1,920 – 1,775 Ma) Orogens that broadly overlapped both temporally and spatially (Rosa et al. 2023; Guarnieri et al. 2023). The pseudo-triangular shape of the body of outcropping rocks of the Mârmorilik Formation (Fig. 1a, b) is the result of two compressional events associated with tectonic inversion of normal faults (Grocott and McCaffrey 2017; Guarnieri and Baker 2022); a first NNE-SSW-oriented tectonic stacking associated with Rinkian deformation followed by NW–SE-oriented compression and tectonic inversion along NE-SW-oriented normal faults with Nagssugtoqidian deformation (Guarnieri et al. 2023).

### An Orosirian evaporite-carbonate platform on an extended passive margin of the Archean Rae Craton

The Paleoproterozoic Mârmorilik Formation in the Karrat Basin hosts the Black Angel Zn-Pb deposit in anhydrite-bearing (2 to 20 vol.% anhydrite) limestone (now present as marble; Rosa et al. 2023). Near the deposit, the host marble with up to 1 wt.% of laths of carbonaceous material (possibly graphite; Rosa et al. 2023) is interpreted as former dolostone, and the ores are capped by graphitic, cherty and pyritic/pyrrhotitic pelitic horizons (Fig. 1c; Guarnieri et al. 2022a; Rosa et al. 2023). The extensive, shallow-marine, evaporite-carbonate platform of the Mârmorilik Formation was host to a significant “brine factory” that charged the deep basin with highly saline fluids (Rosa et al. 2023). Evidence in support of the existence of former evaporite include chlorine-rich scapolite, zones with vuggy porosity and quartz nodules in the ore-bearing marble that are thought to represent metamorphosed, vanished, and replaced evaporites, respectively (Rosa et al. 2023).

Sulfide mineralization is systematically closely associated with anhydrite. The latter in the ca. < 1,950–1,900 Ma Mârmorilik Formation records sulfur isotope ratios (δ^34^S values between + 5.2 and + 12.6‰ – VCDT; Rosa et al. 2023) that are close to (1) the δ^34^S values of ca. 2,160–2,100 Ma carbonate-associated sulfate (CAS; δ^34^S values between + 7.0 and + 14.0‰) deposited during the ca. 2,220–2,060 Ma Lomagundi Carbon Excursion (Planavsky et al. 2012) and (2) evaporites deposited earlier during the Rhyacian (2,300–2,050 Ma; δ^34^S values between + 5.0 and + 18.0‰) (Crockford et al. 2019). Deposition of such evaporites followed a second stage of evolution of ocean chemistry in the Paleoproterozoic (Farquhar et al. 2010; Poulton and Canfield 2011; Planavsky et al. 2011, 2018) as a result of the GOE and transient organic carbon burial during the Lomagundi Carbon Excursion with a time-limited release of atmospheric oxygen (Planavsky et al. 2012; Lyons et al. 2014; Chen et al. 2022). In this context, oceanic sulfate concentrations were at low millimolar levels (Habicht et al. 2002; Farquhar et al. 2010). Thus, the Mârmorilik Formation, which is interpreted as having been deposited in a restricted, epicontinental sea setting on a cratonic margin (Rosa et al. 2023), may be signaling fixation of atmospheric oxygen as sulfate in evaporite after an intermediate state of dissolved seawater sulfate.

## Methodology

### Samples

**Fig. 3:**
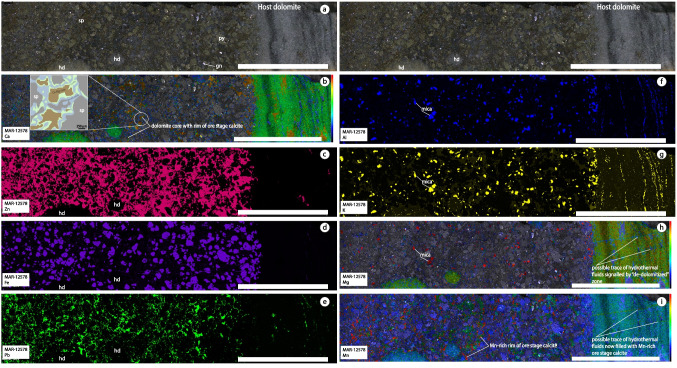
**a**. High-resolution macrophotograph scan of a mineralized drill core section showing the interface between the host dolomite and epigenetic mineralization. **b** to **i**. High-resolution XRF elemental maps (Ca, Zn, Fe, Pb, Al, K, Mg and Mn) of this section of drill core. The inset in Fig. 3**b** shows the textures of dissolution of the host dolomite (found as remaining, brown “Ca-Mg core”), separated from the ore-stage Mn-rich calcite (blue) by an intermediate zone of Ca ± Mg carbonate (light green). Figures 3**b**, **h** and **i** are qualitative intensity maps. All scale bars are 2 cm long. Abbreviations: hd: host dolomite; sp: sphalerite; py: pyrite; gn: galena

All samples utilised in this study come from a collection hosted at the Geological Survey of Denmark and Greenland (GEUS). Our sample set comprises sub-samples of drill cores, outcrop samples and legacy samples from the Black Angel mine. The samples have different modal proportions of sphalerite-pyrite-galena in clean reactive carbonate whereas the massive pyrite samples were found at the contact with pelitic and organic-rich mudstone atop Zn-Pb mineralization (Fig. 3b, c; Tables 1 and 2). As only sulfide amenable to dating by rhenium-osmium (Re-Os) isotope geochemistry in the sulfide paragenesis at Black Angel, pyrite mineral separates (n = 5; Table 1) were produced for Re-Os isotope geochemistry work according to the protocol by Saintilan et al. (2020). The same mineral separates were used for sulfur stable isotope analysis (δ^34^S in ‰ VCDT). This workflow using 70–200 mesh size fractions combines the stepwise use of a Frantz Isodynamic Separator (FIS) and, additional treatment by heavy liquid separation of the magnetic (M) and non-magnetic (NM) fractions obtained at a given current. Pyrite was collected in the NM1.7 fraction. The side slope of the FIS was adjusted to 2º and the NM1.7 fraction was further treated at 1.7 amp in order to remove trace sphalerite and galena into the NM_2_1.7 fraction. Quality control of the final mineral separates was conducted according to the protocol by Saintilan et al. (2020). Five additional pyrite samples were processed to prepare additional mineral separates for Zn stable isotope work for a total of 10 pyrite mineral separates for δ^66^Zn analyses. Along the production of pyrite mineral separates, ca. 10–15 mg of sphalerite were aliquoted from specific samples (n = 6) for δ^66^Zn analyses.

**Table 1:** All Re-Os geochronological data and δ^34^S values for pyrite mineral separates presented in the paper

*With "*^*185*^*Re + *^*190*^*Os spike"*																	
**Sample ID**	**Weight** **(mg)**	**Re** **ng g**^**−1**^	** ± 2σ**	^**187**^**Re** **ng g**^**−1**^	** ± 2σ**	**Total Os** **pg g**^**−1**^	** ± 2σ**	^**192**^**Os** **pg g**^**−1**^	** ± 2σ**	^**187**^**Re/**^**188**^**Os**	** ± 2σ**	^**187**^**Os/**^**188**^**Os**	** ± 2σ**	**rho**	**%Re**_**blk**_	**%**^**187**^**Os**_**blk**_	**%**^**188**^**Os**_**blk**_
MAR 12774-U	488.92	0.749	0.001	0.471	0.000	23.84	0.31	3.24	0.02	459	3	15.73	0.15	0.746	0.77	0.16	3.99
MAR 12774-L	408.77	3.104	0.002	1.951	0.001	85.41	1.18	8.57	0.05	721	5	24.00	0.23	0.666	0.22	0.05	1.85
MAR 12578–1	438.83	3.994	0.001	2.510	0.001	120.42	0.93	14.75	0.04	539	2	18.30	0.07	0.673	0.16	0.03	1.01
MAR 12578–2	389.85	4.067	0.002	2.556	0.001	123.44	1.19	15.47	0.06	523	2	17.72	0.11	0.598	0.18	0.04	1.08
MAR 2085	524.18	0.261	0.000	0.164	0.000	6.75	0.60	0.54	0.03	961	59	31.98	2.59	0.764	2.06	0.45	18.88
*With "*^*185*^*Re + *^*188*^*Os + *^*190*^*Os spike"*																	
**Sample ID**	**Weight** **(mg)**	**Re** **pg g**^**−1**^	** ± 2σ**	^**187**^**Re** **pg g**^**−1**^	** ± 2σ**	^**187**^**Os** **pg g**^**−1**^	** ± 2σ**	**Age** **(Ma)**	** ± 2σ** **(Ma)**	** ± 2σ incl. λ** **(Ma)**							
MAR Massive pyrite	563.06	834	5	524	3	16.21	0.11	1828	16	17							
**Sample ID**	**δ**^**34**^**S** **(‰)**	**2SD**	**n**														
MAR 12774-U	0.68	0.38	3														
MAR 12774-L	2.25	0.46	3														
MAR 12578	3.65	0.16	3														
MAR 2085	3.64	0.44	3														
MAR Massive pyrite	4.08	0.22	3														

Note: *rho* is the error correlation factor of the isotopic ratios

**Table 2:** Zinc isotope composition of pyrite and sphalerite in the Zn-Pb orebody and the overlying massive pyrite cap horizon at Black Angel. These data are used to model the Zn isotope composition of hydrothermal fluids and overall source of Zn. Based on the reference data for Zn isotope compositions of lithologies in Archean craton (Doucet et al. 2018) and our estimate of *δ*^66^Zn values for Mârmorilik carbonate, we compute the respective contribution of the Archean basement and Mârmorilik carbonates in Zn in the orebody. Our estimate of *δ*^66^Zn values for Mârmorilik carbonate is calculated as follows: Sedimentary carbonates are systematically characterized by heavy Zn isotopic signatures (*δ*^66^Zn_marine carbonates_ =  + 0.91 ± 0.47‰, Fig. 5; Pichat et al. 2003; Liu et al. 2016; Dong and Wasylenki 2016). A positive Zn isotopic fractionation between calcite and seawater in modern systems is known with heavy *δ*^66^Zn in calcite in the range + 0.53 to + 1.11‰, with calcite-seawater fractionation factor (*δ*^66^Zn_calcite-seawater_) at ca. 0.60‰ at near neutral pH (Zhao et al. 2021). In largely anoxic oceans in the Paleoproterozoic, oxide-bound Zn burial was limited. Given that burial of oxide-bound Zn is associated with a positive Zn isotope effect, seawater *δ*^66^Zn in the Paleoproterozoic should have remained high in a context of low oxic surface oceans (Isson et al. 2018). Black shales deposited in ancient anoxic settings provide a robust record of the dissolved *δ*^66^Zn values for seawater (*δ*^66^Zn_seawater_) through Earth history (Isson et al. 2018). Thus, *δ*^66^Zn_seawater_ at the time of deposition of the Mârmorilik Formation may have been in the range of *δ*^66^Zn of the sulfidic black shale record ca. 1,900–1,800 Ma (+ 0.40 to + 0.60‰, Fig. 5; Isson et al. 2018). Considering this range of values for *δ*^66^Zn_seawater_ and a positive fractionation factor of + 0.60‰ between seawater and calcite (Zhao et al. 2021), the carbonate-evaporite platform at Maarmorilik could have hosted carbonates with *δ*^66^Zn values at ca. + 1.00 to + 1.20‰ (Fig. 5), i.e., values in the range of documented *δ*^66^Zn values for Pleistocene sedimentary carbonates (Pichat et al. 2003)

Sample	Mineral	Mineralization type	δ^66^Zn (‰)	± 2σ	δ^66^Zn_hydrothermal fluid_ (‰)	± 2σ	δ^66^Zn_source_ (‰)	X = fraction of Zn contributed by Rae Craton basement δ^66^Zn_Rae Craton_ = + 0.30 to + 0.44‰	Y = fraction of Zn contributed by Mârmorilik carbonate δ^66^Zn_carbonate_ = + 1.00 to + 1.20‰
					δ^66^Zn_fluid_ = δ^66^Zn_sphalerite_ + 0.36		δ^66^Zn_source_ = δ^66^Zn_fluid_ – 0.20	*average* = + *0.37‰*	*average* = + *1.10‰*
MAR-12774U	pyrite	sphalerite-pyrite ± galena	0.30	0.03	0.66	0.12	0.46	88%	12%
MAR-12774L	pyrite	sphalerite-pyrite ± galena	0.33	0.04	0.69	0.13	0.49	84%	16%
MAR-12578	pyrite	sphalerite ≥ pyrite ± galena	0.33	0.04	0.69	0.13	0.49	84%	16%
MAR-367902	pyrite	pyrite-sphalerite ± galena	0.35	0.04	0.71	0.13	0.51	81%	19%
MAR-367908	pyrite	sphalerite ≥ pyrite ± galena	0.31	0.04	0.67	0.13	0.47	87%	13%
MAR-367921	pyrite	sphalerite-pyrite ± galena	0.39	0.03	0.75	0.12	0.55	76%	24%
MAR-367934	pyrite	sphalerite-pyrite ± galena	0.36	0.03	0.72	0.12	0.52	80%	20%
MAR-2304	pyrite	pyrite-sphalerite ± galena	0.35	0.03	0.71	0.12	0.51	81%	19%
*Average* ± *2SD (n* = *8)* = + *0.34* ± *0.06‰*									
MAR-2085	pyrite	pyrite-galena ± sphalerite	0.21	0.05	n.a	-	n.a	n.a	n.a
MAR-Masspy	pyrite	massive pyrite above ZnS ore	0.14	0.06	n.a	-	n.a	n.a	n.a
*Average* ± *2SD (n* = *10)* = + *0.31* ± *0.15‰*									
MAR-12578	sphalerite	sphalerite ≥ pyrite ± galena	0.36	0.03	0.72	0.12	0.52	80%	20%
MAR-12774L	sphalerite	sphalerite-pyrite ± galena	0.36	0.03	0.72	0.12	0.52	80%	20%
MAR-12774U	sphalerite	sphalerite-pyrite ± galena	0.36	0.03	0.72	0.12	0.52	80%	20%
MAR-367902	sphalerite	pyrite-sphalerite ± galena	0.39	0.03	0.75	0.12	0.55	76%	24%
MAR-367908	sphalerite	sphalerite ≥ pyrite ± galena	0.34	0.03	0.70	0.12	0.50	83%	17%
MAR-367978	sphalerite	sphalerite ≥ pyrite ± galena	0.34	0.04	0.70	0.13	0.50	83%	17%
*Average* ± *2SD (n* = *6)* = + *0.36* ± *0.04‰*									

n.a.: not applicable

The supplementary material describes the detailed methodology used in the present work including (1) mineralogical investigations using reflected/transmitted light microscopy of polished sections of the samples utilized for pyrite Re-Os geochronology and zinc stable isotope work, and micro-XRF analysis of a flat drillcore section, procedures for (2) pyrite Re-Os isotope geochemistry by isotope dilution and negative thermal ionization mass spectrometry (ID-N-TIMS) of purified Re and Os aliquots, (3) bulk pyrite sulfur stable isotope (δ^34^S), and (4) pyrite (n = 10) and sphalerite (n = 6) zinc stable isotope geochemistry (δ^66^Zn).

## Results

### Sulfide mineralization and its host rock

Macrophotographs of a drillcore section of a representative pyrite-sphalerite ± galena-mineralized sample are presented along with XRF elemental maps for calcium (Ca), Zn, iron (Fe), Pb, aluminum (Al), potassium (K), magnesium (Mg), and manganese (Mn) (Fig. 3). The contact between the host dolomite and the Zn-Pb-Fe-mineralized section is irregular and consistent with a replacement style of mineralization (Fig. 3a and b). Remnants of the host dolomite (< 1 cm-large clots – hd) are identified in amongst sulfides (Fig. 3a, b, c, d, e). Sphalerite ± galena cement subhedral pyrite grains. Linear mineralized features with sphalerite ± galena follow black seams in the host dolomite (right-hand side of Fig. 3a, c, e). Coarse-grained to fine-grained Mg-bearing mica (likely phlogopite) is identified in the mineralized section and the host rock dolomite, respectively, based on Al, K and Mg elemental maps (Fig. 3f, g, h). There is a clear difference in grain-size of mica between the sulfide-bearing section and the host dolomite (i.e., smaller grains in the non-mineralized zone; Fig. 3f and g). The systematic association of mica with sulfide mineralization is discussed later.

In the host dolomite, there are discordant veinlets filled with manganese(Mn)-rich ore-stage calcite (Fig. 3i). The Mg elemental map reveals a pattern of lower Mg contents in these discordant features compatible with de-dolomitization during hydrothermal fluid flow and precipitation of Mn-rich ore-stage calcite (Fig. 3h and i). The combination of the Ca, Mg and Mn elemental maps (Fig. 3b, h, i) shows that the remaining clots of host dolomite in amongst sulfides have: (1) a Mg- and Ca-rich core compatible with the host dolomite; (2) an intermediate zone with lesser Mg contents; and (3) a Mn-rich rim at the contact with sulfides, sphalerite in particular (inset in Fig. 3b). We address the importance and significance of this Mn-rich ore-stage calcite with respect to interpreting and identifying the source of hydrogen sulfide for mineralization.

**Fig. 4:**
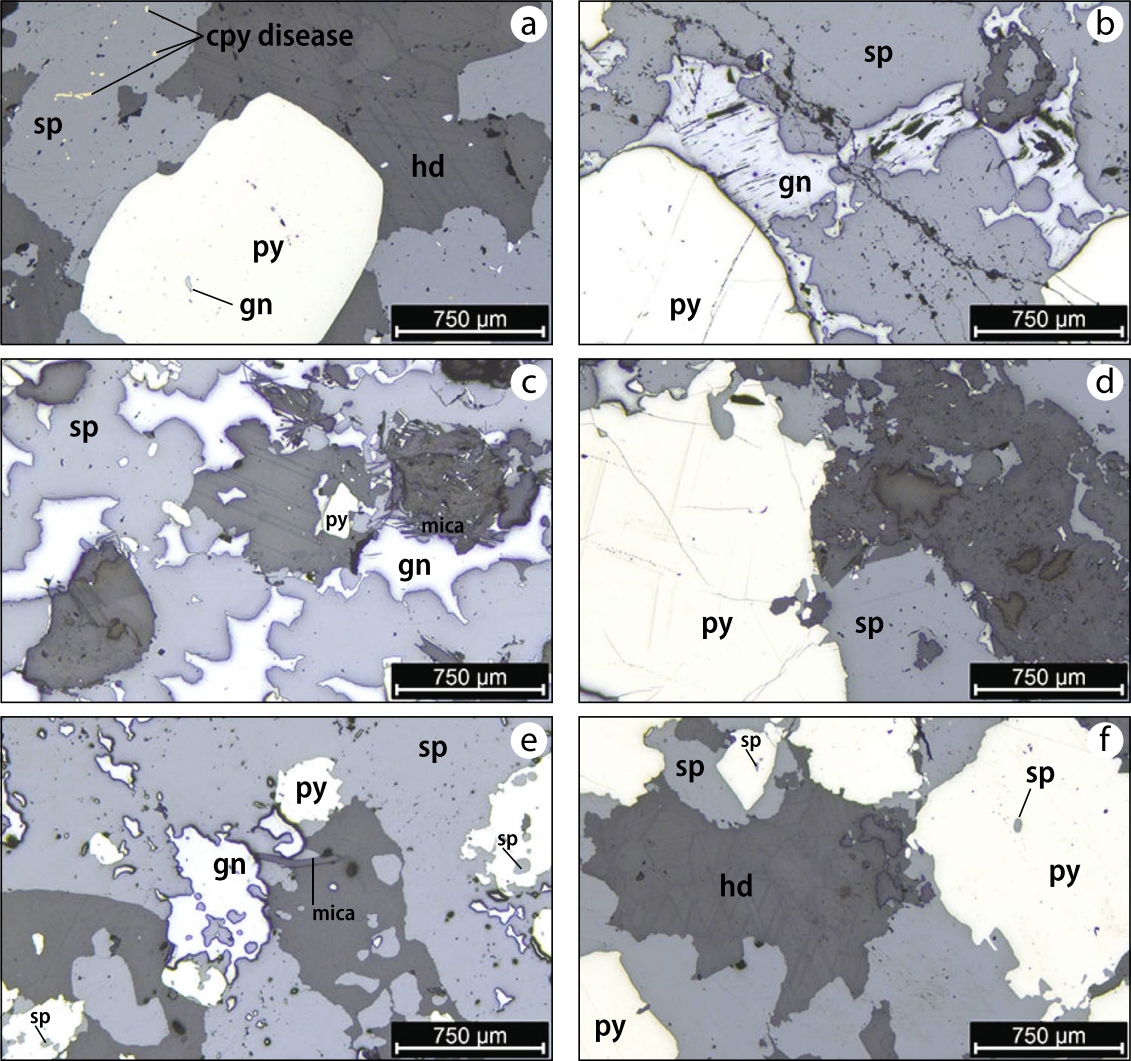
Paragenetic relationships in reflected light microscopy between hydrothermal dolomite, pyrite and Zn-Pb mineralization. Panels **a-d** correspond to three of the four samples utilized for pyrite Re-Os geochronology that constrains a Re-Os isochron age for MVT mineralization ca. 1,884 Ma. Panels **e & f** show paragenetic relationships of pyrite and Zn-Pb sulfides in representative samples (MAR-367902, MAR-367908) for the suite of additional samples utilized for Zn stable isotope geochemistry. Note: chalcopyrite diseased in sphalerite in panel **a**. Abbreviations: sp: sphalerite, gn: galena, py: pyrite, cpy: chalcopyrite, hd: hydrothermal dolomite

Large (> 1 mm) subhedral and smaller (< 500 μm) anhedral grains of pyrite, galena and sphalerite replaced the host dolomite (Fig. 4). Inclusions of sphalerite and galena in pyrite signal some precipitation of galena and sphalerite coeval with pyrite formation (Fig. 4a, e, f). Yet, the whole sulfide assemblage is approximately coeval. Laths of hydrothermal mica in rosette-like texture are intimately associated and intergrown with, primarily galena (Fig. 4c), but to a much lesser extent with sphalerite (Fig. 4c and e).

### Re-Os isotope data

A first run of Re-Os isotope geochemistry using aliquots of the “^185^Re + ^190^Os spike” equilibrated with aliquots of each pyrite mineral separates signaled that massive pyrite (sample MAR-1) is bereft of common Os whereas all other pyrite samples (n = 4) contain common Os (Table 1). Therefore, a new aliquot of MAR-1 was digested with a known mass of “^185^Re + ^188^Os + ^190^Os spike” in order to accurately and precisely quantify the total content of radiogenic ^187^Os*. Pyrite in sample MAR-1 has low Re (834 ± 5 pg g^−1^) and very low radiogenic ^187^Os* (16.21 ± 0.11 pg g^−1^) contents. Its ^187^Re-^187^Os model age is 1,828 ± 16 [17] Ma (2σ = 0.9% total uncertainty; bracketed uncertainty includes the 0.31% uncertainty in the decay constant of ^187^Re after Smoliar et al. 1996).

**Fig. 5:**
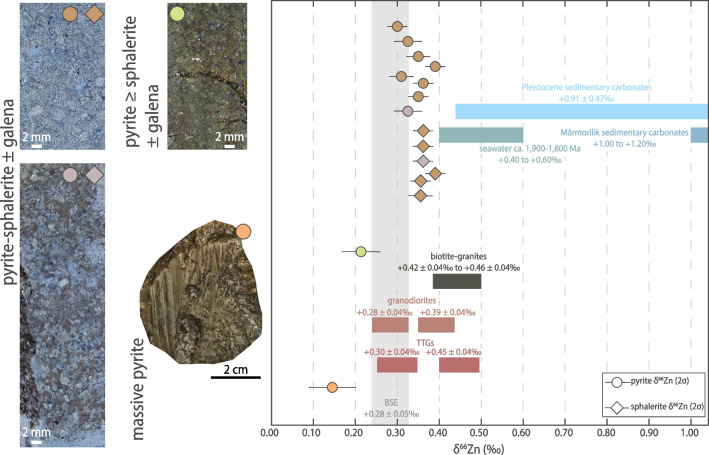
Representative samples of the sulfide mineralization at Black Angel and corresponding Zn isotope composition (δ^66^Zn_Lyon-JMC_) of pyrite and sphalerite. Reference Zn isotope values (1) for crystalline basement rocks after Doucet et al. (2018), (2) for Paleoproterozoic seawater after Isson et al. 2018, and (3) estimated Zn isotope values for Mârmorilik sedimentary carbonate (estimates in this study using data from Isson et al. (2018) and Zhao et al. (2021)). Range of Zn isotope values for Pleistocene carbonates after Pichat et al. (2003)

**Fig. 6:**
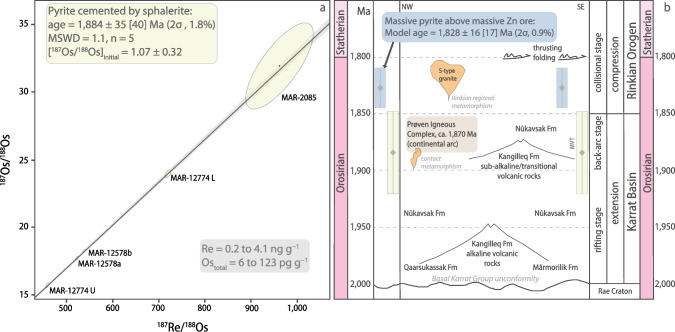
a. Model 1 five-point isochron regression of data points for pyrite in conventional ^187^Os/^188^Os vs. ^187^Re/^188^Os diagram (see text for details). Individual ellipses show 2σ uncertainty of each data point in ^187^Os/^188^Os vs. ^187^Re/^188^Os space. Ellipses are constructed from maximum and minimum error vectors that are orthogonal to each other. Maximum and minimum uncertainties are statistical values that are calculated from uncertainties of the ^187^Os/^188^Os and ^187^Re/^188^Os ratios for a given data point. Final uncertainties were calculated by full error propagation of uncertainties in the Re and Os measurements, blank values, isotopic compositions, and reproducibility of the standard Re and Os values. **b**. Tectonic event chart summarizing the evolution of the Karrat Basin and the Rinkian Orogen (modified from Guarnieri et al. 2023). The new Re-Os date of isochron regression in Fig. 6a and the model date of massive pyrite in the graphitic mudstone cap rock are shown with full analytical uncertainty, including uncertainty of the decay constant of ^187^Re (see Smoliar et al. 1996)

Pyrite in the four pyrite-sphalerite ± galena-mineralized samples (e.g. in Fig. 5) has low to moderate Re, and low Os contents (0.261 to 4.067 ng g^−1^ Re; 6.7 to 120.4 pg g^−1^ Os). The ^187^Re/^188^Os ratios are high (523–961) and positively correlated with highly radiogenic ^187^Os/^188^Os ratios (15.7–32.0) in the ^187^Os/^188^Os vs. ^187^Re/^188^Os space (Fig. 6a). The Re-Os data for these pyrite mineral separates yield a Model 1 date of 1,884 ± 35 [40] Ma (Model 1 computed by IsoplotR, Vermeesch 2018; n = 5; mean square of weighted deviates – MSWD = 1.1; 2σ = 1.8% total uncertainty; initial ^187^Os/^188^Os ratio – Os_i_ = 1.07 ± 0.32).

### Sulfur stable isotope data

The sulfur isotopic composition (δ^34^S-VCDT) of the five mineral separates utilized for Re-Os isotope geochemistry work is presented in Table 1. Each reported average and its standard deviation (SD) are based on three aliquots per sample. Pyrite in the four pyrite-sphalerite ± galena-mineralized samples returns δ^34^S values between 0.68 ± 0.19‰ and 3.64 ± 0.22‰, respectively. In contrast, massive pyrite possesses δ^34^S values at 4.08 ± 0.11‰.

### Zinc stable isotope data

Sulfide mineral-specific (pyrite or sphalerite) Zn isotopic values (δ^66^Zn) are reported in Table 2. The δ^66^Zn values of all samples range from + 0.14 ± 0.06‰ to + 0.39 ± 0.03‰ (2σ), with pyrite (n = 10) and sphalerite (n = 6) having average (± 2 standard deviation – 2SD) at + 0.31 ± 0.15‰ and + 0.36 ± 0.04‰, respectively. Yet, except for two pyrite aliquots, all other sphalerite and pyrite aliquots have identical δ^66^Zn values within uncertainty. Pyrite δ^66^Zn values in pyrite-sphalerite ± galena samples range from + 0.30 ± 0.03‰ to + 0.39 ± 0.03‰ whereas sphalerite δ^66^Zn values in pyrite-sphalerite ± galena samples range from + 0.34 ± 0.03‰ to + 0.39 ± 0.03‰ (Fig. 5). The two exceptions are: (i) pyrite in pyrite-galena ± sphalerite mineralization with a δ^66^Zn signature at + 0.21 ± 0.05‰ (sample MAR-2085), and (ii) massive pyrite in the cap horizon above the zinc ore body with a δ^66^Zn value of + 0.14 ± 0.06‰.

## Discussion

### A MVT deposit formed in back-arc setting after a first inversion of the Paleoproterozoic rift

#### Pyrite Re-Os isochron age of the sphalerite-galena mineralizing event

**Fig. 7:**
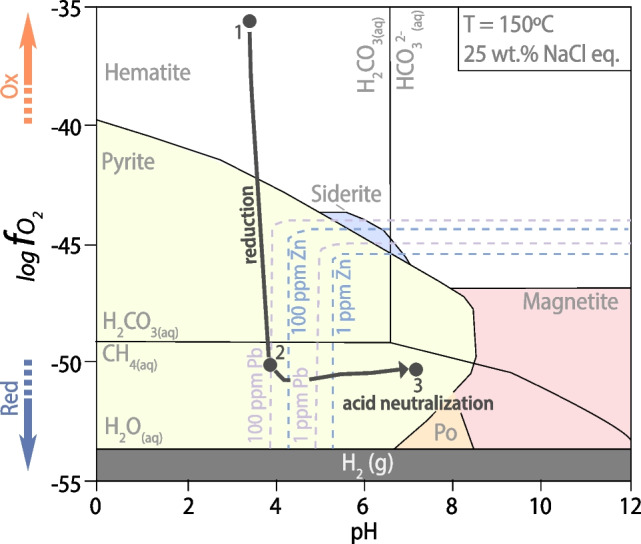
Reaction path in Eh–pH diagram at 150 °C and 25 wt.% NaCl eq. (modified from Liu et al. 2021) leading to reduction and acid neutralization of a metal-bearing, brine-derived hydrothermal fluids as those responsible for mineralization at Black Angel

We document a Model 1 Re-Os isochron date of 1,884 ± 35 [40] Ma for pyrite in pyrite-sphalerite ± galena mineralization (Fig. 6a). However, the Black Angel Zn-Pb mineralization underwent greenschist-facies (T < 500 °C; Pedersen 1980) metamorphic events resulting in intense multi-stage deformation and remobilization of sulfide minerals (Garde 1978; Pedersen 1980, 1981; Horn et al. 2019; Rosa et al. 2023), and this metamorphic impact on Re-Os systematics in pyrite must be considered. The Re-Os geochronometer in pyrite is a robust and mature geochronometer (N_robust ages_ > 20; Rooney et al. 2024) with knowledge from mineralogical studies about the residency of Re and Os in pyrite (Hnatyshin et al. 2020) and the closure temperature of the Re-Os system at ca. 600 °C in this sulfide (Brenan et al. 2000; Hnatyshin et al. 2020; Rooney et al. 2024). Given that the metamorphism did not exceed 600 °C, the Re-Os systematics in pyrite was not disturbed nor reset despite subsequent greenschist-facies metamorphic overprint. Therefore, our new pyrite Re-Os isochron date can be interpreted as a robust and geologically meaningful age for epigenetic Zn-Pb mineralization in “chemically reactive” (Fig. 7) clean carbonate units.

The paragenetic relationships (Figs. 3 and 4) indicate a sequence of mineralization replacing the host dolomite as follows: (1) precipitation of subhedral to anhedral pyrite with minute inclusions of sphalerite and/or galena, (2) followed by galena accompanied by hydrothermal mica, and (3) extensive sphalerite precipitation. This sequence of mineralization is compatible with an Eh–pH evolution of metal-bearing brines that would undergo reduction (path 1 to 2 in Fig. 7, adapted after Liu et al. 2021) and sustained acid neutralization converging to sphalerite precipitation (path 2 to 3 in Fig. 7), with galena accompanying pyrite and sphalerite. The peculiar relationship of galena with rosettes of hydrothermal mica signals the paramount requirement of acid neutralization to switch to extensive sphalerite mineralization (path 2 to 3 in Fig. 7). In conclusion, a general take-home message is that pyrite in such a sequence of mineralization with sphalerite and galena can be used to effectively date Zn-Pb mineralization by pyrite Re-Os geochronology supported by detailed petrographic investigations. Similar conclusions were reached for the use of pyrite Re-Os dating to constrain the onset and duration of Zn-Pb mineralization in the Irish Zn-Pb ore field (Hnatyshin et al. 2015).

#### Geodynamic trigger and tectonic context of Zn-Pb mineralization

At 1,884 ± 35 Ma, the ca. < 1,950–1,900 Ma platform carbonates of the Mârmorilik Formation were located in (1) a back-arc setting that experienced emplacement of the Prøven igneous complex magmatic arc in connection with the Rinkian Orogen, and (2) in the far foreland of the Nagssugtoqidian Orogen (Figs. 1, 2b and 6b; Guarnieri et al. 2023). Thus, the 1,884 ± 35 [40] Ma timestamp constrains the timing of Zn-Pb mineralization at Black Angel to the following:

- During far-field fluid flow (i.e., basin-derived hydrothermal fluids) associated with back-arc spreading (ca. 1,900 – 1,850 Ma), and in response to the first inversion of the Paleoproterozoic rift starting from ca. 1,900 Ma (Figs. 2a, b and 6b), and;
- Before the development of the Rinkian foreland basin (ca. 1,850 – < 1,800 Ma) and a collisional stage (ca. 1,830 – < 1,800 Ma) in the context of the telescoping Rinkian (ca. 1,900 – < 1,800 Ma; Fig. 2c, d; Guarnieri and Baker 2022; Guarnieri et al. 2022a, c, 2023) and the Nagssugtoqidian Orogens (ca. 1,920 – 1,775 Ma; Kalsbeek et al. 1987; Connelly et al. 2000; Grocott and McCaffrey 2017; Rosa et al. 2023; Guarnieri et al. 2023).

Therefore, MVT mineralization at Black Angel (Fig. 2e) preceded thrusting and folding of thin-skinned tectonic units above the foreland starting ca. 1,800 Ma (Figs. 2b-d and 6b). We propose that back-arc spreading was the driving and geothermal force needed for the flow of basin-derived, hydrothermal fluids to favourable trap sites in the Mârmorilik Formation where reduced sulfur was available for Zn-Pb sulfide mineralization. In fact, this geodynamic context of formation of a carbonate-hosted, epigenetic Zn-Pb mineralization related to far-field fluid flow associated with back-arc spreading is akin that for Paleozoic MVT deposits in the Canadian Cordillera (Nelson et al. 2002).

On the other hand, the Re-Os model age (1,828 ± 16 Ma) of the massive pyrite body at the contact with the (now pelitic) organic-rich (now possibly graphitic; Rosa et al. 2023), relatively carbonate-poor, and less reactive mudstone cap rock above the Zn-Pb orebodies (Fig. 1c and inset; Fig. 2e) is interpreted as signaling a new event of pyrite precipitation. The latter is coeval with a geodynamic context of collision and tectonic compression between ca. 1,830 and < 1,800 Ma at Black Angel (Fig. 2c, d). Similar massive pyrite bodies were documented on the edges of the Cretaceous Zn-Pb-sulfide orebodies (‘mantos’) in the San Vicente MVT mine (Central Peru) (Spangenberg et al. 1999). These compact lenses of pyrite, which are pseudomorphs after marcasite (i.e., itself compatible with acidic conditions at pH < 5.5), were interpreted as a sign of cessation of pH increase of the mineralizing fluid, which triggered the massive precipitation of iron disulfide instead of sustaining sphalerite precipitation at higher pH (Fig. 7; Spangenberg et al. 1999). In fact, at San Vicente, compressional tectonic events were shown to be coeval with only episodic fluid circulation that did not result in MVT Zn-Pb mineralization (Tavazzani et al. 2024). Therefore, by analogy with the San Vicente MVT orebodies, we suggest that: (1) at ca. 1,828 Ma, in a geodynamic context of collision and tectonic compression, fluid circulation was only episodic at Black Angel and the hydrologic system was barren, whereas (2) like the Triassic rift at San Vicente (Tavazzani et al. 2024), the first inversion of the rift hosting the Karrat Basin at Maarmorilik/Black Angel was the fertile event with a hydrologic system favourable to Zn-Pb sulfide precipitation ca. 1,884 Ma. We speculate that the hydrologic system became unfavourable for mineralization by ca. 1,828 M because of: (1) the lack of chemical reactivity of the mudstone cap rock, and (2) the unfavorable impact of incipient regional Rinkian metamorphism (ca. 1,830–1,800 Ma; Kokfelt et al. 2023; Grocott et al. 2023; Guarnieri et al. 2023) on aquifer permeability and continued brine migration (Figs. 2c and 6b).

#### Source of reduced sulfur for MVT mineralization

The identification of the geodynamic trigger and context for epigenetic Zn-Pb mineralization in the present work contextualizes previous findings (e.g., radiogenic Pb isotopes; Taylor and Kalsbeek 1990; Partin et al. 2021, and in situ sulfide δ^34^S values; Partin et al. 2021) to decipher mineralizing processes at Maarmorilik. All data collectively support the model of MVT mineralization recently proposed for the Black Angel mineral system (Rosa et al. 2023). The remnants of dolomite in the Zn-Pb-Fe-sulfide mineralized zones (Figs. 3 and 4) are compatible with host rock dissolution by acidic metal-bearing brine-derived hydrothermal fluids (Rosa et al. 2023) that are typically involved in MVT mineralization (Leach et al. 2010). Another line of evidence signaling the presence of acidic hydrothermal fluids during mineralization is the common association of hydrothermal mica with galena (Fig. 4c and e).

The source of reduced sulfur for MVT mineralization needs to be documented to complete our present genetic model. Hydrogen sulfide consumed in MVT mineralization is produced either by bacterial or thermochemical sulfate reduction (BSR vs. TSR; Spangenberg and Macko 1998; Spangenberg et al. 1999; Machel 2001; Spangenberg and Herlec 2006; Herlec et al. 2010; Cai et al. 2022). The graphitic, pelitic cap rock that hosts the massive pyrite cap layer above the Maarmorilik Zn-Pb orebody is intepreted as a seal and stratigraphic barrier (Fig. 1c and inset; Fig. 2e) that could have focused hydrocarbon gas and reduced sulfur compounds (e.g., hydrogen sulfide) into the underlying trap of the reactive carbonate units (Rosa et al. 2023). In the presence of hydrocarbons and post-diagenetic temperatures, TSR is the most likely pathway of reduction of aqueous sulfate (Spangenberg and Macko 1998; Machel 2001; Herlec et al. 2010; Saintilan et al. 2016; Cai et al. 2022). TSR generally takes place at temperatures within the range of 120 to 200 °C, with fractionation between aqueous sulfate and hydrogen sulfide being limited in the range of 1.0 to 3.0‰, i.e., Δ^34^S[H_2_S-SO_4-aq_] = –[1–3]‰ (Machel et al. 1995). Given the metamorphic overprint on mineralization, fluid inclusion microthermometry of primary fluid inclusion assemblages is impeded. Therefore, information about the original temperature of mineralization remains speculative (Partin et al. 2021), possibly below 250 °C (Rosa et al. 2023). We consider the δ^34^S values of anhydrite at Maarmorilik (+ 5.2 to + 12.6‰ Rosa et al. 2023) as the best available and representative proxy of seawater sulfate δ^34^S values at Maarmorilik in the Paleoproterozoic. Thus, our bulk δ^34^S values for pyrite (+ 0.7 to + 4.1‰; Table 1) and published in situ δ^34^S values for pyrite (+ 3.4 to + 4.5‰), sphalerite (+ 0.9 to + 6.1‰) and galena (+ 0.0 to + 4.2‰; Partin et al. 2021) are compatible with a bimodal source of reduced sulfur: (1) a deep-seated source of reduced sulfur probably related to the leaching of magmatic sulfides (δ^34^S values at 0.3 ± 0.5‰; Seal 2006) in basement rocks by saline brines, and (2) TSR-driven production of H_2_S (calculated δ^34^S values: + 2.2 to + 9.6‰) for sulfide mineralization at Maarmorilik. We propose that a TSR mechanism for the supply of H_2_S is further supported by the presence of Mn-rich ore-stage calcite either in discordant linear features of de-dolomitization (Fig. 3i) or rimming de-dolomitized remnants of the host dolomite around a Ca-Mg-rich core in the Zn sulfide mineralization (inset in Fig. 3b). Ore-stage Mn-rich calcite is a characteristic by-product of oxidation of organic compounds by MnO_2_ during diagenesis, at the same time when aqueous sulfate is being reduced into H_2_S by TSR (Okita and Shanks 1992). An open question after this study and that of Rosa et al. (2023) remains the nature and origin of organic compounds that would have been present in the aquifer at Maarmorilik for TSR to take place.

### Bimodal source of Zn for mineralization at Black Angel

Within the established geodynamic context of a MVT mineralizing system at Black Angel, we can identify the source(s) of metals for mineralization by combining our new Os and Zn isotope data with previous knowledge brought by Pb isotope studies. The initial ^187^Os/^188^Os ratio (Os_i-pyrite_ = 1.07 ± 0.32) in our isochron regression of data points for pyrite in pyrite-sphalerite ± galena mineralization is much higher than estimates for the contemporaneous upper mantle (Os_mantle-1,884 Ma_ = 0.12 ± 0.01, 1σ; estimate after Meisel et al. 2001) and the average upper continental crust at the time of mineralization (Os_UCC-1,884 Ma_ = 0.16 ± 0.12, 1σ; estimate after Chen et al. 2016). This Os_i-pyrite_ clearly signals a radiogenic crustal source of Os. This source must have been sufficiently old with moderate to high Re/Os to accumulate radiogenic Os from the time of its formation until pyrite precipitation ca. 1,884 Ma. Lead isotope compositions of galena in three orebodies at Black Angel are compatible with derivation of Pb from a homogeneous and local crustal source within the Karrat Basin with a Pb isotopic composition matching that of ca. 3,100 to 2,800 Ma orthogneisses in the Rae Craton (Taylor and Kalsbeek 1990; Connelly and Thrane 2005; Partin et al. 2021). Such lithologies in the ca. 3,150–2,660 Ma Rae Craton, including in the immediate vicinity of the Maarmorilik locality (Thrane 2021), stand as highly probable sources of radiogenic crustal Os in the Maarmorilik orebody and, by corollary, other metals (see Saintilan et al. 2021) such as Zn according to the following rationale. Cratons (including the Rae Craton; Thrane 2021) are generally comprised of orthogneisses and tonalite-trondhjemite-granodiorite-suite (TTGs) granitoid intrusions (50–70% of cratonic shields) with possible presence of biotite-granites resulting from melting of felsic rocks (Moyen and Laurent 2018; Hoffmann et al. 2019 and references therein). In these Archean cratons, average Zn contents are: 22–70 ppm Zn in TTGs, 21–120 ppm Zn in (grano-)diorites (“sanukitoids”), and 28–30 ppm Zn in biotite-granites (Doucet et al. 2018). Leaching experiments confirm that crystalline basement rocks act as metal sources for hydrothermal sulfide deposits (Goldhaber et al. 1995; Leach et al. 2005), with Pb and Zn released by felsic minerals, with additional contribution of Zn by hydrothermal alteration of biotite (Burisch et al. 2016).

In addition to the main source of Pb in the basement rocks of the Rae Craton, it was speculated that a complementary source of Pb in the Maarmorilik limestone (now marble) might have been involved to explain some Pb isotope data of galena at Maarmorilik (Partin et al. 2021). Specifically, excess thorogenic ^208^Pb in sulfides in MVT deposits hosted in carbonate aquifers was explained by derivation of Pb from sedimentary limestone upon ore-related hydrothermal recrystallization into dolomite (Goldhaber et al. 1995). Thus, sedimentary carbonates have been envisaged as a source of metals in specific conditions (e.g., for Zn in Kelley et al. 2009; Fuji et al. 2011; Wilkinson 2023) because calcite can adsorb and substitute trace elements for Ca^2+^ in its lattice (octahedral site; Dromgoole and Walter 1990; Burton 1993) with preferential substitution by similarly coordinated metal cations like Zn^2+^, Cu^2+^ or Fe^2+^ (Parekh et al. 1977; Reeder 1996; Reeder et al. 1999; Morse et al. 2007). In the following, we explore the possibility of a supplementary source of Zn in the host limestone considering our new Zn isotope data for pyrite and sphalerite (Fig. 5 and Table 2). Zinc isotope values for sphalerite and pyrite in the Black Angel deposit are interpreted as pristine from the time of mineral precipitation because it is currently empirically suggested that high-temperature metamorphic overprint does not alter original δ^66^Zn isotopic signatures in sulfides (Peck et al. 2022). Our δ^66^Zn values for sphalerite (+ 0.34 ± 0.03‰ to + 0.39 ± 0.03‰; Fig. 5) are remarkably uniform (average =  + 0.36 ± 0.04‰; Table 2). Pyrite in the pyrite-sphalerite ± galena zones also has remarkably uniform δ^66^Zn values (average =  + 0.34 ± 0.06‰; Table 2; Fig. 5). Conversely, the δ^66^Zn values of the younger ca. 1,828 Ma massive pyrite sample (+ 0.14 ± 0.06‰) in the graphitic, pelitic cap rock horizon (Fig. 1c and inset, Fig. 2e), and the underlying pyrite ≥ sphalerite ± galena sample (+ 0.21 ± 0.05‰) may be interpreted as signaling precipitation at isotopic disequilibrium and fractionation in relatively carbonate-poor units where acid neutralization is not sufficient to promote sphalerite precipitation. We conclude that our data with δ^66^Zn values for sphalerite would illustrate a single stage mineralizing event at ca. 1,884 Ma with neutralization of acidity and efficient precipitation of sphalerite at equilibrium consequent to dissolution of clean, chemically reactive carbonate. Similar conclusions were drawn from Proterozoic clastic-dominated Zn-Pb deposits (e.g., McArthur River, northern Australia) in which the invariant δ^66^Zn signature of the ores is consistent with efficient, near-quantitative metal precipitation from Zn-saturated fluids (Baumgartner et al. 2021). Our interpretation also supports the prediction (Leach et al. 2005, 2010) that the economic potential of MVT deposits in Proterozoic carbonates depends on the extent of sphalerite precipitation that is controlled by the reactivity of carbonate lithologies for acid neutralization and pH increase (Fig. 7; see also Liu et al. 2021). We favor this interpretation because a sequential precipitation of sphalerite in space and time would leave a pattern of Rayleigh isotopic fractionation with lighter Zn isotopes preferentially incorporated in the earliest and most proximal sphalerite to precipitate (Wilkinson et al. 2005; Kelley et al. 2009; Wilkinson 2023). This is not observed in the present study.

**Fig. 8:**
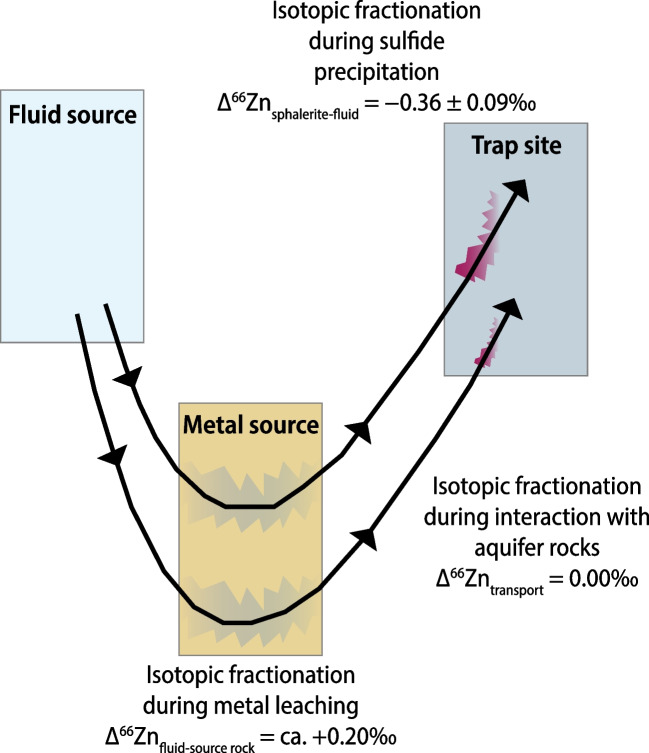
General concept of Zn isotope fractionation from source to sink for the fluid flow of metal-bearing hydrothermal fluids (modified from Wilkinson 2023). The various estimates of isotope fractionation factors are from: (1) Fernandez and Borrok (2009) & Mathur and Wang (2019) for isotope fractionation during metal leaching; (2) Pons et al. (2013) for isotope fractionation during fluid flow; (3) Archer et al. (2004) for isotope fractionation during sphalerite precipitation

In light of the previous sections, we contend that Zn was supplied dominantly from crystalline rocks in the basement comprising the Archean Rae Craton, and in a complementary way by the dolomitized limestone host to the Black Angel deposit. To ascertain this statement, we use the known fractionation factors (Fig. 8) to stepwise back-calculate an estimate for the Zn isotope composition of the composite source of metals from the analyzed δ^66^Zn values of sphalerite (n = 6) and pyrite (n = 8, except samples MAR-2085 and MAR-masspy; Fig. 5, Table 2) at isotopic equilibrium in the present work (Table 2). To this end, we successively calculate (1) an estimate of the δ^66^Zn value of the hydrothermal fluid from which sulfides precipitated by utilizing the Δ^66^Zn_sphalerite-fluid_ factor (–0.36 ± 0.09‰; Archer et al. 2004; see note about the case of pyrite in Table 2), and then (2) the bulk δ^66^Zn value of a composite source of Zn undergoing fluid-rock interaction by using a Δ^66^Zn_fluid-rock_ estimate at + 0.20‰ (Table 2; Fernandez and Borrok 2009; Pons et al. 2013). The composite Zn source would have a δ^66^Zn composition between + 0.46 and + 0.55‰ (Table 2), i.e., higher than the δ^66^Zn values of crystalline basement rocks (according to the database by Doucet et al. 2018; Fig. 5). Our estimate permits calculation and modelling of the respective contributions in Zn from the crystalline rocks in the Rae Craton (average δ^66^Zn signature =  + 0.37‰; Table 2) and from the host dolomitized limestone (estimated average δ^66^Zn_Maarmorilik sedimentary carbonate_ =  + 1.10‰; see Table 2 for details; Fig. 5) according to the formula:

1 $${X}_{\%Rae Craton}= \frac{\left(\delta {}^{66}{Zn}_{source}-\delta {}^{66}{Zn}_{M\hat{a} rmorilik limestone}\right)}{\left(\delta {}^{66}{Zn}_{Rae Craton}-\delta {}^{66}{Zn}_{M\hat{a} rmorilik limestone}\right)}$$

$${Y}_{\text{\%}Maarmorilik limestone }=100 -{X}_{\text{\%}Rae Craton};$$

Our modelling indicates that, at minimum, ca. 76 to 88% of total Zn was supplied in the Black Angel deposit by hydrothermal fluids leaching crystalline basement rocks in the Rae Craton. In addition, dissolution of the host dolomitized Maarmorilik carbonate supplied the remaining ca. 12 to 24% of total Zn (Table 2). Such an accessory source of Zn in sedimentary calcite is deemed possible in the overall geological and ecological context of the Paleoproterozoic. In Paleoproterozoic seawater at high Na/Cl ratio (Shalev et al. 2018), Zn concentrations were similar to modern concentrations, with hydrothermal fluxes of Zn on the seafloor maintaining high levels of dissolved Zn in the surface ocean (Scott et al. 2013). In the absence of Zn-based eukaryotic metabolisms before ca. 1,700–1,600 Ma and their ecological dominance in the Neoproterozoic (Scott et al. 2013; Isson et al. 2018), the precipitation of halite-gypsum in the Maarmorilk evaporite-carbonate platform (Rosa et al. 2023) would have been concomitant with a local decrease of chloride concentration in low-temperature Paleoproterozoic surface seawater. At low chloride concentration, incorporation of isotopically heavy Zn in sedimentary carbonate via adsorption-incorporation mechanisms (Zachara et al. 1991; Paquette and Reeder 1995; Watson 1996; Temmam et al. 2000; Elzinga and Reeder 2002; Dong and Wasylenki 2016; Smrzka et al. 2019) would have been possible at Maarmorilik. This sedimentary calcite sink would have been a complementary sink to the main sink of Zn identified in black shale (Isson et al. 2018). This context and the loops and feedback between the biosphere and the lithosphere would explain the presence of isotopically heavy Zn in the Paleoproterozoic Maarmorilik limestone, and its availability for Zn-Pb mineralization at Black Angel.

## Conclusions

Pyrite Re-Os geochronology coupled with mineral-specific (pyrite and sphalerite) Zn and S isotope analysis in the Zn-Pb Black Angel deposit (Greenland) reveal that Black Angel formed ca. 1,884 Ma as the product of a Paleoproterozoic MVT mineralizing system in platform carbonates. The mineralizing processes were related to far-field hydrothermal fluid flow associated with back-arc spreading (ca. 1,900 – 1,850 Ma) in response to the first inversion of a Paleoproterozoic rift starting from ca. 1,900 Ma. Back-arc spreading was the driving and geothermal force needed for the flow of basin-derived, hydrothermal fluids to favourable trap sites where reduced sulfur was available for Zn-Pb sulfide mineralization. In fact, in addition to the Paleozoic MVT deposits in the Canadian Cordillera (Nelson et al. 2002) and the Mesozoic San Vicente MVT deposit (Peru; Spangenberg et al. 1999; Tavazzani et al. 2024), our new findings document a third example of an accurately and precisely dated MVT mineralizing system that is genetically related to a geodynamic context of back-arc spreading.

Base metals at Black Angel were derived from a crustal source inferred from the highly radiogenic initial Os isotopic signature in pyrite associated with sphalerite and galena. Our new sulfide-specific Zn isotope data identify a bimodal source of metals with an accessory supracrustal source in addition to the commonly accepted and dominant source of metals in basement rocks of the Rae Craton (ca. 75–90% of Zn). The supracrustal accessory source of Zn (ca. 10 – 25% of Zn) comprises sedimentary calcite in Paleoproterozoic platform carbonates. We interpret the alteration of calcite to dolomite as the reason for release of Zn from calcite and its subsequent recapture in sphalerite. This source of Zn in the sedimentary host rock is deemed possible in the context of Paleoproterozoic seawater at high Na/Cl ratio and in the absence of Zn-based eukaryotic metabolism in shallow marine environment.

The results from our study suggest several criteria required to determine prospectivity for MVT Zn-Pb deposits in Paleoproterozoic carbonate rocks:

- Suitable platform carbonates with chemically reactive clean dolomitized limestone that were deposited in a sag basin that transitioned to a rifting stage;
- Epigenetic sulfide Zn-Pb mineralization that predates all tectonic features related to the collisional stage and thin-skinned tectonics (fold-and-thrust system) and deformation;
- Sources of metals from crystalline basement and supplementary source of Zn from dolomitized limestone;
- Determination early in exploration activities of absolute time constraints from sulfide Re-Os geochronology to place the timing of mineralization within the temporal framework of the geodynamic evolution of the study area.

## Supplementary Information

Below is the link to the electronic supplementary material.

Supplementary file1 (DOCX 23 KB)

## Data Availability

All data produced in this study are available in the present manuscript and its data tables.
