# Supplementary material for: Paleoproterozoic Mississippi Valley-type mineralization at Black Angel, Greenland: evidence from sulfide δ66Zn and rhenium-osmium geochronology
Source: Miner Depos. 2024 Dec 9;60(5):1039–57. doi: 10.1007/s00126-024-01332-w (PMC12065769; doi:10.1007/s00126-024-01332-w)
Supplement: Supplementary file 1 — Supplementary file1 (DOCX 23 KB) [file 126_2024_1332_MOESM1_ESM.docx]

**Paleoproterozoic Mississippi Valley-type mineralization at Black Angel, Greenland: evidence from sulfide δ^66^Zn and rhenium-osmium geochronology**

Nicolas J. Saintilan^1,5^, Corey Archer^1^, Kristoffer Szilas^2^, Kristina Krüger Geertsen^2^, Diogo Rosa^3^, and Jorge E. Spangenberg^4^

^1^Institute of Geochemistry and Petrology, Department of Earth Sciences, ETH Zürich, Zürich, Switzerland

^2^Department of Geosciences and Natural Resource Management, University of Copenhagen, Copenhagen, Denmark

^3^Department of Mapping and Mineral Resources, Geological Survey of Denmark and Greenland (GEUS), Copenhagen, Denmark

^4^Institute of Earth Surface Dynamics, University of Lausanne, Lausanne, Switzerland

^5^Present address: Department of Geological Sciences, University of Alabama, Box 870338, Tuscaloosa 35487, AL, United States of America

**Supplementary Material: Methods**

**Mineralogical investigations**

Samples were analyzed using a Bruker Tornado M4+ micro-XRF instrument (Bruker Nano GmbH, Berlin) at the Department of Geosciences and Natural Resource Management, University of Copenhagen. Micro-XRF analysis is non-destructive and enables the fast determination of elements and their distribution on a flat sample surface (Flude et al. 2017). This micro-XRF instrument uses a Rh X-ray source with a polycapillary lens that focuses the X-rays to a 20 µm spot size. The resultant X-ray signal is captured with two XFlash® silicon drift detectors (130 eV resolution at Mn-Kα; Bruker 2023). The measured samples were analyzed under low-vacuum conditions (2 mbar) to avoid Ar absorption and thus improve the detection of lighter elements. The Rh X-ray tube energy was set at 50 kV and a current of 600 µA. Measurements were performed using a 20 μm step size and an acquisition time of 20 ms per pixel. Spectral quantification was performed in standardless mode using the Tornado M4 software (version 1.6). The resulting energy intensity maps show the distribution of selected elements, and are presented as two-dimensional image files (Figs. 5a-i). In addition, polished sections of key samples utilized for rhenium-osmium and zinc isotope geochemistry (see below) were studied under reflected and transmitted light microscopy to investigate paragenetic relationships between pyrite and sphalerite-galena.

**Rhenium-osmium (Re-Os) isotope geochemistry**

For each analysis, between 389 and 563 mg of pyrite mineral separates were weighed and transferred into thick-walled borosilicate Carius tubes (Shirey and Walker, 1995). Each sulfide aliquot was dissolved in inverse Aqua Regia (~3 mL of 11N HCl and ~6 mL 16N HNO_3_) with a known amount of “^185^Re+^190^Os spike” solution at 210ºC for 24h (Laboratory of Rhenium-Osmium Isotope Geochemistry and Geochronology, Isotope Geochemistry and Cosmochemistry Group, Institute of Geochemistry and Petrology, ETH Zürich). The laboratory protocol used in the present work is described in full details in (Selby and Creaser, 2001; Selby et al., 2009; Hnatyshin et al., 2016; Li et al., 2017). In brief, Os was isolated and purified from the inverse Aqua Regia solution by chloroform (CHCl_3_)-hydrobromic acid solvent extraction at room temperature, and, by microdistillation (Roy-Barman and Allègre 1995; Cohen et al. 1996; Shen et al. 1996; Birck et al. 1997; Selby and Creaser 2001). The Re was isolated using an acetone-sodium hydroxide (Acetone-NaOH) step (Matthews and Riley 1970; Bozkhov et al. 1985; Li et al. 2009), followed by HCl–HNO_3_-based anion chromatography (Morgan et al. 1991; Cumming et al. 2013). The Re and Os isotope compositions were determined by negative thermal ionization mass spectrometry (N-TIMS) using a Thermo Scientific Triton mass spectrometer at the Institute of Geochemistry and Petrology, ETH Zürich. Rhenium and Os were loaded onto outgassed Ni and Pt filaments, respectively. Rhenium was measured as ReO_4_^–^ in static mode on Faraday collectors, whereas Os was measured as OsO_3_^–^ in peak-hopping mode on a single electron multiplier (Creaser et al. 1991; Völkening et al. 1991). Measurement quality was monitored by repeated measurements of in-house Re (125 pg aliquot – ^185^Re/^187^Re = 0.59833 ± 0.00067, 2σ, n = 22) and Os (Durham Romil Os Standard, DROsS; Nowell et al. 2008; 50 pg aliquot – ^187^Os/^188^Os = 0.16092 ± 0.00069, 2σ, n = 12) standard solutions. Total procedural blank using inverse Aqua Regia was 2.82 ± 0.06 pg Re, 170 ± 9 fg Os with a blank ^187^Os/^188^Os isotope composition of 0.62 ± 0.05 (2σ, n = 2). The analytical uncertainties result from full error propagation of weighing errors, spike calibration, standard measurements, mass spectrometry analyses and blanks.

**Bulk sulfur stable isotope (δ^34^S)**

Sulfur isotope measurements were performed at the Institute of Earth Surface Dynamics of the University of Lausanne via elemental analysis-isotope ratio mass spectrometry (EA/IRMS) with a system composed of a Carlo Erba 1108 elemental analyzer equipped with an AS-200LS pneumatic autosampler connected to a Delta V Plus isotope ratio mass spectrometer via a ConFlo III interface, as described previously (Spangenberg et al. 2010, 2022). The stable isotope composition of sulfur is reported in the delta (δ) notation as the per mil (‰) deviation of the isotope ratio relative to known standards: δ = [(R_sample_ - R_standard_)/R_standard_], where R is the ratio of the heavy to light isotopes (^34^S/^32^S). The sulfur standard is the Vienna Cañon Diablo Troilite (VCDT). The reference SO_2_ gas was calibrated against the IAEA-S-1 sulfur isotope reference standard (Ag_2_S) with δ^34^S value of –0.3‰. The normalization of the measured δ^34^S values to the VCDT scale and the assessment of the overall analytical reproducibility of the EA/IRMS analyses were performed by replicate analyses of laboratory standards (UNIL-pyrite-E, –6.72‰ and UNIL-cinnabar, 15.82‰) and international reference materials (RMs) IAEA-S-1, IAEA-S-2, and IAEA-S-3 silver sulfides (–0.3‰, 22.62±0.16‰, and –32.49±0.16‰, respectively) (Spangenberg et al. 2022). The overall analytical reproducibility of the EA/IRMS analyses was better than ±0.3‰ (1 SD). The accuracy of the δ^34^S analyses was checked periodically by analyses of RM.

**Zinc stable isotope geochemistry (δ^66^Zn)**

Zinc stable isotope geochemistry (protocol by Archer and Vance 2004) was carried out on sub-aliquots of 10 pyrite mineral separates and 6 sphalerite sub-aliquots (Table 2). Isotopic analyses were performed using a Thermo-Finnigan NeptunePlus multicollector inductively coupled mass spectrometer (MC-ICP-MS) at the Institute of Geochemistry and Petrology, ETH Zürich. Zinc was introduced into the mass spectrometer in 0.3 M HNO_3_ via a PFA nebulizer (50 μl min^-1^) attached to an Aridus. Mass discrimination was corrected using the double spike, as detailed by Bermin et al. (2006). All Zn isotopic compositions are given in standard notation as follows relative to the JMC Lyon Zn standard:

$$\delta{}^{66}\mathrm{Zn}=1000\left[ \frac{(\frac{{}^{66}\mathrm{Zn}}{{}^{64}{Zn)sample}}}{(\frac{{}^{66}\mathrm{Zn}}{{}^{64}\mathrm{Zn})Lyon JMC}}-1 \right]$$

Long-term reproducibility of isotopic analyses was assessed over the course of this and parallel studies, through repeated measurements of the AA-ETH Zn isotopic standard (Archer et al. 2017) calibrated against the JMC Lyon standard as well as a secondary standard for Zn (IRMM-3702). These latter give δ^66^Zn_Lyon-JMC_ = +0.30 ± 0.06‰.

**References**

Archer C, Vance D (2004) Mass discrimination correction in multiple-collector plasma source mass spectrometry: an example using Cu and Zn isotopes. J. Anal. At. Spectrom. 19:656– 665.

Archer C, Andersen MB, Cloquet C, Conway TM, Dong S, Ellwood M, Moore R, Nelson J, Rehkämper M, Rouxel O, Samanta M, Shin KC, Sohrin Y, Takano S, Wasylenki L (2017) Inter-calibration of a proposed new primary reference standard AA-ETH Zn for zinc isotopic analysis. J. Anal. At. Spectrom 32:415–419.

Bermin J, Vance D, Archer C, Statham PJ (2006) The determination of the isotopic composition of Cu and Zn in seawater. Chem. Geol. 226:280-297.

Birck JL, Roy-Barman M, Capmas F (1997) Re-Os isotopic measurements at the femtomole level in natural samples. Geostandard Newsletter, 21:19-27.

Bozkhov OD, Jordanov N, Borissova LV, Fabelinskii YI (1985) Extraction-spectral emission determination of traces of rhenium using ICP. Fres. Zeitschr. Anal. Chem., 321:453-456.

Bruker (2023). Lab Report XRF 456. Analysis of geological thin sections

Cohen AS, Waters FG (1996) Separation of osmium from geological materials by solvent extraction for analysis by thermal ionisation mass spectrometry. Anal. Chim. Acta, 332:269-275.

Creaser RA, Papanastassiou DA, Wasserburg GJ (1991) Negative thermal ion mass spectrometry of osmium, rhenium and iridium. Geochim. Cosmochim. Acta, 55:397**–**401.

Cumming VM, Poulton SW, Rooney AD, Selby D (2013) Anoxia in the terrestrial environment during the late Mesoproterozoic. Geology, 41:583-586.

Flude S, Haschke M, Storey M, Harvey J (2017) Application of benchtop micro-XRF to geological materials. Mineralogical Magazine, 81:923-948.

Hnatsyhin D, Kontak DJ, Turner EC, Creaser RA, Morden R, Stern RA (2016) Geochronologic (Re-Os) and fluid-chemical constraints on the formation of the Mesoproterozoic-hosted Nanisivik Zn-Pb deposit, Nunavut, Canada: Evidence for early diagenetic, low-temperature conditions of formation. Ore Geol. Rev. 79:189-217.

Li C, Qu W, Du A, Sun W (2009) Comprehensive study on extraction of rhenium with acetone in Re-Os isotopic dating. Rock Miner. Anal., 28:233-238 (in Chinese, with English abstract).

Li Y, Selby D, Condon D, Tapster S (2017) Cyclic magmatic-hydrothermal evolution in porphyry systems: High-precision U-Pb and Re-Os geochronology constraints on the Tibet Qulong porphyry Cu-Mo deposit. Econ. Geol. 112:1419-1440.

Matthews A, Riley J (1970) The determination of rhenium in seawater. Anal. Chim. Acta, 51:483-488.

Morgan JW, Golightly DW, Dorrzapf AF (1991) Methods for the separation of rhenium, osmium and molybdenum applicable to isotope geochemistry. Talanta, 38:259-265.

Nowell GM, Pearson DG, Parman SW, Luguet A, Hanski E (2008) Precise and accurate ^186^Os/^188^Os and ^187^Os/^188^Os measurements by multi-collector plasma ionisation mass spectrometry, part II: Laser ablation and its application to single-grain Pt-Os and Re-Os geochronology. Chem. Geol., 248:394-426.

Roy-Barman M, Allègre CJ (1995) ^187^Os/^186^Os in oceanic island basalts: tracing oceanic crust recycling in the mantle. Earth Planet. Sci. Lett., 129:145-161.

Selby D, Creaser RA (2001) Re-Os geochronology and systematics in molybdenite from the Endako porphyry molybdenum deposit, British Columbia, Canada. Econ. Geol., 96:197-204.

Selby D, Kelley KD, Hitzman MW, Zieg J (2009) Re-Os sulphide (bornite, chalcopyrite, and pyrite) systematics of the carbonate-hosted copper deposits at Ruby Creek, southern Brooks Range, Alaska. Econ. Geol., 104:437–444.

Shen JJ, Papanastassiou DA, Wasserburg GJ (1996) Precise Re-Os determinations and systematics of iron meteorites. Geochim. Cosmochim. Acta, 60:2887-2900.

Shirey SB, Walker RJ (1995) Carius tube digestion for low-blank rhenium-osmium analysis. Anal. Chem., 67:2136–2141.

Spangenberg JE, Lavrić J, Meisser N, Serneels V (2010) Sulfur isotope analysis of cinnabar from Roman wall paintings by EA/IRMS –tracking the origin of archaeological red pigments and their authenticity. Rapid Comm. Mass Spec. 24:2812-2816.

Spangenberg JE, Saintilan NJ, Palinkas SS (2022) Safe, accurate, and precise sulfur isotope analyses of arsenides, sulfarsenides, and arsenic and mercury sulfides by conversion to barium sulfate before EA/IRMS. Anal Bioanal Chem. 414:2163-2179.

Völkening J, Walczyk T, Heumann K (1991) Osmium isotopic ratio determination by negative thermal ionization mass spectrometry. Inter. J Spectro. Ionic Phys., 105:147**–**159.
